# Dorsal visual stream and *LIMK1*: hemideletion, haplotype, and enduring effects in children with Williams syndrome

**DOI:** 10.1186/s11689-023-09493-x

**Published:** 2023-08-26

**Authors:** J. Shane Kippenhan, Michael D. Gregory, Tiffany Nash, Philip Kohn, Carolyn B. Mervis, Daniel P. Eisenberg, Madeline H. Garvey, Katherine Roe, Colleen A. Morris, Bhaskar Kolachana, Ariel M. Pani, Leah Sorcher, Karen F. Berman

**Affiliations:** 1 Section on Integrative Neuroimaging, Intramural Research Program, National Institute of Mental Health, National Institutes of Health, Bethesda, MD 20892 USA; 2 Clinical and Translational Neuroscience Branch, Intramural Research Program, National Institute of Mental Health, National Institutes of Health, Bethesda, MD 20892 USA; 3 Neurodevelopmental Sciences Laboratory, Department of Psychological and Brain Sciences, University of Louisville, Louisville, KY 40202 USA; 4 Department of Pediatrics, Kirk Kerkorian School of Medicine at UNLV, Las Vegas, NV 89102 USA; 5 Department of Biology, University of Virginia, Charlottesville, VA 22903 USA

**Keywords:** Williams syndrome, Hemideletion, *LIMK1*, Dorsal stream, Intraparietal sulcus

## Abstract

**Background:**

Williams syndrome (WS), a rare neurodevelopmental disorder caused by hemizygous deletion of ~ 25 genes from chromosomal band 7q11.23, affords an exceptional opportunity to study associations between a well-delineated genetic abnormality and a well-characterized neurobehavioral profile. Clinically, WS is typified by increased social drive (often termed “hypersociability”) and severe visuospatial construction deficits. Previous studies have linked visuospatial problems in WS with alterations in the dorsal visual processing stream. We investigated the impacts of hemideletion and haplotype variation of *LIMK1*, a gene hemideleted in WS and linked to neuronal maturation and migration, on the structure and function of the dorsal stream, specifically the intraparietal sulcus (IPS), a region known to be altered in adults with WS.

**Methods:**

We tested for IPS structural and functional changes using longitudinal MRI in a developing cohort of children with WS (76 visits from 33 participants, compared to 280 visits from 94 typically developing age- and sex-matched participants) over the age range of 5–22. We also performed MRI studies of 12 individuals with rare, shorter hemideletions at 7q11.23, all of which included *LIMK1*. Finally, we tested for effects of *LIMK1* variation on IPS structure and imputed *LIMK1* expression in two independent cohorts of healthy individuals from the general population.

**Results:**

IPS structural (*p* < 10^−4^ FDR corrected) and functional (*p* < .05 FDR corrected) anomalies previously reported in adults were confirmed in children with WS, and, consistent with an enduring genetic mechanism, were stable from early childhood into adulthood. In the short hemideletion cohort, IPS deficits similar to those in WS were found, although effect sizes were smaller than those found in WS for both structural and functional findings. Finally, in each of the two general population cohorts stratified by *LIMK1* haplotype, IPS gray matter volume (*p*_discovery_ < 0.05 SVC, *p*_replication_ = 0.0015) and imputed *LIMK1* expression (*p*_discovery_ = 10^−15^, *p*_replication_ = 10^−23^) varied according to *LIMK1* haplotype.

**Conclusions:**

This work offers insight into neurobiological and genetic mechanisms responsible for the WS phenotype and also more generally provides a striking example of the mechanisms by which genetic variation, acting by means of molecular effects on a neural intermediary, can influence human cognition and, in some cases, lead to neurocognitive disorders.

**Supplementary Information:**

The online version contains supplementary material available at 10.1186/s11689-023-09493-x.

## Background

Although recent decades have witnessed a remarkable wealth of gene discovery, elucidating the functional roles of identified genes and gene networks in the development and maintenance of biological processes and the mechanisms by which genetic alterations contribute to human phenotypes and diseases remains a central challenge. Without the option of experimentally manipulating the genomes of living humans, more nuanced methods based on combined evidence from experiments in model organisms and hypothesis-driven analyses of mutations arising naturally in humans can provide strategies for such gene-based “biological discovery.” Investigating genomic mechanisms of human disorders affecting higher cognitive function has been particularly difficult because of the limitations of animal models for many complex human characteristics and the concomitant problem of extrapolating to the context of the human brain. Studying individuals with rare, highly penetrant mutations constitutes a particularly powerful approach for discerning the neurobiological roles played by specific genes in typical human function and for discovering potential molecular pathways for novel treatments targeting conditions in which such functioning is disrupted.

Williams syndrome (WS), a rare neurodevelopmental disorder (OMIM 194050), offers a unique opportunity in this regard by virtue of its combination of a well-delineated genetic abnormality and a well-characterized behavioral and cognitive profile. Genetically, WS is caused by a hemizygous deletion of ~1.6 megabases from chromosomal band 7q11.23, known as the Williams syndrome critical region (WSCR) which harbors ~25 genes. The affected segment of DNA is flanked by chromosome-specific low-copy repeat sequences with high sequence homology and recurrent genomic rearrangements of this chromosomal region, resulting in an unusually stereotyped WS hemideletion with similar breakpoints occurring in more than 95% of affected individuals [1]. Clinically, WS is typified by remarkable social drive (often termed “hypersociability”) and a unique profile of higher cognitive functions, the hallmark of which is severe weakness in visuospatial construction. The WS cognitive phenotype is highly sensitive and specific in delineating WS [2–4], and previous studies have linked the visuospatial deficits found in WS with alterations in the structure and function of the dorsal visual processing stream, a fundamental neural circuit that is one of two primary processing pathways of the primate visual cortex [5]. Reductions in neural activation during cognition [6], sulcal depth/gyrification [7–9], and gray matter volume [6, 10, 11] within the dorsal steam’s intraparietal sulcus (IPS) region, as well as reductions in IPS functional connectivity [12–14], have all been consistently reported and replicated in the literature. Although the IPS undoubtedly plays a role in several brain systems, lesion [15] and activation [16, 17] studies have demonstrated that this region, particularly in the right hemisphere [16, 17], is especially relevant to the types of visuospatial abilities most affected in WS.

Despite the considerable degree to which the brain and behavioral features of WS have been documented [18] and the detail in which the molecular structure of the WS copy number variation has been characterized, it is not known how or which of the ~25 genes hemideleted in WS contributes to this neurobehavioral and brain phenotype. Previous studies have identified potential neurological influences of several WS genes, including *DNAJC30*, [19] *FZD9*, [20] *GTF2I*, [21–23] LIMK1 [13], and *STX1A*, [24] with a particularly compelling neurobiological mechanism involving myelin, brain structure, and social behavior identified in a *GTF2I* knockout mouse model [21]. While haploinsufficiencies of a number of 7q11.23 genes likely interact during development to produce the brain and behavioral characteristics of WS, LIM domain kinase 1 (*LIMK1*) may play a particularly important role, since it is preferentially expressed in the brain [25] and its protein product is involved, via the phosphorylation of cofilin, in the regulation of actin, a protein that plays a primary role in the subcellular machinery that drives neuronal migration [26, 27]. In the complex spatiotemporal orchestration of interactions between a cell’s membrane and its extracellular matrix that enables directed cell motion, actin filaments are a key component of the “molecular clutch” that engages and disengages in order to accomplish local membrane motion [28]. The radial unit model [29] describes how proper brain development critically depends on appropriate neuronal migration along transient glial-cell scaffolding within the fetal cerebral wall, and Van Essen’s tension-based theory of morphogenesis [30] implies that improper neuronal migration could lead to anomalies in cortical folding such as those found in the IPS of individuals with WS [7, 8]. Additionally, Limk1 knockout mouse studies [31–33] have demonstrated abnormal dendritic spine morphology at the cellular level as well as impaired spatial learning and altered fear response at the behavioral level. Previous work has identified a functional haplotype of three *LIMK1* single-nucleotide polymorphisms (SNPs rs6460071, rs710968, and rs146777179) that lie in the promoter region (near the 5′ end) of *LIMK1* and appear to impact transcription [34].

Taken together, these preclinical studies lend plausibility to the hypothesis that genetic variations in *LIMK1* could produce measurable effects on dorsal stream gray matter in humans, and, thus, that haploinsufficiency of this gene plays an important role in the cognitive and brain phenotype in WS. In further support of this possibility, a previous study of two rare families with smaller hemideletions in 7q11.23 has linked the WS cognitive profile to the *LIMK1* gene [3]. However, such a role for *LIMK1* has been controversial because some individuals with small deletions that include *LIMK1* do not show visuospatial constructive impairment [35]. Often lost in the controversy is the fact that genes do not directly code for behaviors per se but, rather, code for molecular processes that act on neurons and neural systems, which, in turn, function as the intermediaries of genetic effects on complex behaviors. In the case of *LIMK1*, for example, a multitude of intervening factors operating at these intermediary levels may serve to modulate its effects on cognition or behavior. The brain is thus more proximally affected by genes than is behavior, and the penetrance of genetic variation at the level of neural structure and function is expected to be higher [36].

## Methods

We took four experimental approaches to test the hypothesis that variation in *LIMK1* affects the IPS in WS. First, we confirmed that the IPS structural and functional anomalies, previously reported largely in adults, are present in children with WS. Second, with longitudinal imaging (structural brain scans acquired at 76 visits over an age range of 5–22 years), we showed that these anomalies are stable from early childhood to adulthood, consistent with an enduring genetic mechanism. Third, to more specifically link these structural and functional features to WS genes, we studied extraordinarily rare individuals having 7q11.23 hemideletions that do not include more telomerically located genes within the WS 7q11.23 locus that are typically hemideleted in WS (see supplementary Fig. S2). Importantly, these “short deletions” (SDs) have in common a hemideletion of *LIMK1* (and elastin, but elastin has minimal expression in human brain parenchyma and is specifically not associated with the WS cognitive phenotype) [3]. Fourth, we tested for associations between a *LIMK1* haplotype previously associated with gene expression and IPS functional connectivity (based on the three *LIMK1* SNPs described above) [14, 34] and cortical organization in a discovery cohort of healthy, well-screened, typically developed members of the general population (GP), and we replicated those findings in a second such cohort. We hypothesized that we would find variations in IPS gray matter structure and/or function associated with *LIMK1* hemideletions (in the SD study) and with *LIMK1* haplotype variation (in the two general population cohorts). Such associations demonstrate the functional relevance of *LIMK1* and might result from developmental alterations in LIMK1 protein availability via hemideletion or SNP-dependent changes in regulated processes such as alternative transcripts and/or splicing.

### Cross-sectional study of structural and functional anomalies in children with Williams syndrome

In our initial cross-sectional investigation, we studied 31 children with WS (mean age 9.2 ± 3.2, 21 females) with typical WSCR deletions who had IQs in the normal to low-normal range. A summary of participant demographics for our initial structural study of children is shown in Table 1. For each participant visit, we acquired three 3-Tesla structural scans (GE MR-750, MEMPRAGE, 124 axial slices, *TR* = 10.5 ms, *TE* = 1.8 ms, resolution 1 × 1 × 1 mm). These images were intensity normalized [37] and then registered and averaged with AFNI [38] tools to improve signal-to-noise ratios. We used SPM12’s tissue segmentation and applied SPM12 [39] tools to perform diffeomorphic warping to a common space (based on a template balanced for age, sex, and group — WS vs. TD) and generate Jacobian-modulated maps of gray matter in the resulting standard space. After smoothing at 8-mm FWHM, we used AFNI’s 3dttest++ to search for voxel-wise gray-matter differences between the children with WS and typically developing children, covarying for age, sex, and total brain volume.

**Table 1:** Demographics of children in initial cross-sectional structural/functional studies

Group	N	Age (years)	Sex (M/F)
WS structural	31	9.2 ± 3.2	10/21
TD structural	64	9.3 ± 1.6	22/42
WS functional	12	11.3 ± 2.3	1/11
TD functional	22	11.6 ± 2.5	13/9

We performed functional MRI imaging on a subset (*N* = 12, mean age 11.3 ± 2.3, 11 females) of the children with WS above, on the same 3-Tesla scanner (GE MR-750). During these sessions, participants played a customized version of the video game Tetris, during which the children tried to fit a puzzle piece into a puzzle “landscape” as the piece descended down the screen. The level of difficulty was parameterized to allow for varying abilities and for flexibility in analyses. Following denoising, slice-timing correction, motion correction, warping to a study-specific template, artifact removal [40], and smoothing at 8-mm FWHM, SPM12 was used to perform general linear modeling to localize regions differentially active across the relevant contrast (“difficult” trials vs. “easy” trials) and between the two participant groups. We performed several post hoc analyses that successfully ruled out potentially problematic influences of group differences in sex ratios, task performance, motor responses, and motion.

### Longitudinal study of structural and functional anomalies in children with Williams syndrome

In our expanded, longitudinal structural study, we scanned 33 children with WS (76 visits, mean visit age 12.0 ± 4.4, sex distribution of visits 23 M/53 F), acquiring (including TD participants) a total of 1056 MEMPRAGE structural MRI scans from 356 visits. Table 2 summarizes participant demographics for our longitudinal study of children with WS, and Fig. S1 depicts the timelines of visits for participants with WS and typically developing participants. Spatial normalization was performed within individuals and then on a group basis. Specifically, scans for all of an individual’s visits were spatially normalized to create a mid-time-point average image for that participant using SPM12’s longitudinal normalization tool [39]. Advanced Normalization Tools (ANTS) software [41] was used to derive a group template that was balanced for age and sex, and then the mid-time-point average image for each participant was warped to this template. Next, for each participant, ANTS was used to concatenate the deformation fields from the two spatial normalization stages, enabling the transformation to be performed with a single interpolation. Gray-matter images were Jacobian modulated based on the composite deformation field from each visit into the common group template space, followed by smoothing at 8-mm FWHM. From these data, voxel-wise penalized-spline models of longitudinal gray-matter trajectories across the brain were created using a generalized additive mixed-model (GAMM) approach (specifying participant as a random effect) as implemented in R’s gamm4 package [42] and AFNI’s 3dMSS tool [43]. We also performed linear mixed-effects cross-sectional analyses with AFNI’s 3dLME [44] to assess voxel-wise gray-matter differences between the groups, covarying for age, sex, and total brain volume.

**Table 2:** Demographics of children in longitudinal structural/functional studies

Group	*N*	Sex (M/F)	Visits	Visits age range (years)	Mean age of visits (years)	Sex distribution of visits (M/F)
WS structural	33	11/22	76	5.7–22.3	12.0 ± 4.4	23/53
TD structural	94	38/56	280	5.5–22.5	12.3 ± 3.2	85/195
WS functional	15	2/13	43	7.5–19.8	14.4 ± 3.4	8/27
TD functional	34	19/15	66	7.8–19.9	13.8 ± 3.2	35/28

For the longitudinal fMRI study, the contrast images (“difficult” trials vs. “easy” trials) from each visit (preprocessed as described above for the cross-sectional study) were used to model voxel-wise penalized-spline-based trajectories and determine group differences as described above for the longitudinal structural study.

All participants in the cross-sectional and longitudinal studies gave informed consent (written consent by parents of minor children and assent by children) according to National Institutes of Health Institutional Review Board guidelines.

### Structural and functional anomalies in adults with short deletions

For the short deletion study, we acquired and analyzed structural and functional MRI scans for 12 individuals (mean age = 35.4 ± 13.7, nine females) ascertained by FISH screening of individuals who were referred with suspected WS and cascade screening of siblings and parents of probands [23]. Deletion breakpoints were localized using SNP copy number analyses with Affymetrix 500 K SNP microarrays and/or real-time PCR as described previously [3, 45], and breakpoints were confirmed by PCR amplification and sequencing of deletion junction fragments when possible. Each participant was determined to have one of five hemideletions that included *LIMK1* (see Fig. S2). We compared their gray matter patterns and task-based fMRI responses to those of carefully matched controls (see Table 3). Gray matter volume was examined using a voxel-based morphometry (VBM) approach, employing diffeomorphic spatial normalization tools [46] to analyze structural MRI scans. For each participant, we acquired six 1.5 Tesla structural scans (SPGR, 124 axial slices, *TR* = 12 ms, *TE* = 5.2 ms, resolution 0.9375 × 0.9375 × 1.2 mm). These images were intensity normalized [37] and then registered and averaged with AFNI [38] tools to improve signal-to-noise ratios. We used SPM8’s tissue segmentation and DARTEL [46] tools to perform diffeomorphic intersubject alignment and generate Jacobian-modulated maps of gray matter in the resulting group-specific space. After smoothing at 6-mm FWHM, we performed an ANCOVA with SPM5 (http://www.fil.ion.ucl.ac.uk/spm/.SPM) to search for voxel-wise gray-matter differences between the groups, covarying for age, sex, and total brain volume.

**Table 3:** Demographics for participants with SD in structural study

	SD (*n* = 12)	Controls (*n* = 12)	*P*-value
Gender	9 F, 3 M	9 F, 3 M	1 (NS)
Age	35.4 ± 13.7	31.5 ± 8.0	0.4 (NS)
IQ	97.0 ± 10.8	97.0 ± 6.4	1 (NS)

Age and IQs are shown as mean ± standard deviation. IQs were measured by the two-subset form of the WASI [47] for participants with SD and a short form of the Wechsler Adult Intelligence Scale-Revised [48] for all except one control (for whom the WASI was used). All these participants were of Caucasian ancestry. One control was left-handed; all other participants were right-handed

The fMRI portion of the study challenged the visuospatial system by asking the participant to determine whether two shapes presented on a screen could be assembled to form a square [6]. This “square completion” task block was contrasted with a “shape-matching” task block, in which participants indicated whether two shapes were identical. Blood oxygen level-dependent T2*-weighted gradient-echo echo-planar images (*TR* = 3 s, *TE* = 30 ms, *FOV* = 24 cm, 90° flip, 64 × 64 matrix, 36 contiguous and sequential slices, voxel size 3.75 × 3.75 × 4 mm) were acquired on a 3-Tesla GE scanner with whole-head coil. Stimuli during scanning consisted of pairs of black shapes presented to the left and right of a fixation cross, with task instruction present throughout the block under the cross. For motor control blocks, each presentation was of a matching pair, and the same button was pressed. During “match” blocks, a shape was presented with either an identical copy or its mirror image. Participants pressed one of two buttons depending on whether or not they were the same. During “square completion,” participants were instructed to determine whether or not the two shapes could be assembled to form a square without flipping them over. For sensorimotor “control” trials, participants were shown two matching forms, repeated across all trials, and were asked to simply press a button at presentation. The three task conditions were presented in 16-s-long blocks, with a 2.8-s inter-trial interval. The task was self-paced, with a maximum response window of 7 s per trial. Performance data are summarized in Table 4. Phase-shifted, motion-corrected functional images were aligned to each individual’s structural images and affine transformed into a study-specific standard space averaged across all healthy controls and SD participants. Event covariates for each condition type (square, match, and control) were entered into a general linear model along with motion and drift covariates of no interest. Random effects analyses were used to localize regions differentially active across the different task conditions and between the two participant groups. All participants gave written informed consent according to National Institutes of Health Institutional Review Board guidelines.

**Table 4:** Demographics and square completion performance for fMRI participants with SDs

	SD (*n* = 10)	Controls (*n* = 12)	*P*-value
Gender	7 F, 3 M	4 F, 8 M	0.2 (NS)
Age	35.1 ± 12.5	33.0 ± 7.7	0.6 (NS)
IQ	97.7 ± 10.8	94.7 ± 6.5	0.4 (NS)
Match accuracy (%)	77 ± 14.9	85 ± 10.6	0.09(NS)
Match reaction time (ms)	1445 ± 177	1432 ± 199	0.9 (NS)
Square accuracy (%)	62 ± 13.1	69 ± 12.0	0.2 (NS)
Square reaction time (ms)	2111 ± 761	2152 ± 530	0.9(NS)

Age, IQ, accuracy, and reaction time are shown as mean ± standard deviation. IQ was measured by the two-subset form of the WASI [47] for participants with SDs and a short form of the Wechsler Adult Intelligence Scale-Revised [48] for controls. One fMRI control was African American; all other participants were of Caucasian ancestry. One control was left-handed; all other participants were right-handed

### General population study: NIMH cohort

We studied a group of 255 healthy, right-handed Caucasian volunteers under the age of 50 years (mean age 33 ± 9.7 [std. dev.]; 113 males, 142 females), all of whom were given a Structured Clinical Interview for DSM-IV (SCID) to rule out the presence of any psychiatric illness, physical and neurological examinations, a battery of neuropsychological tests, and a screening MRI examination. Exclusion criteria included inability to give informed consent, learning disabilities, confounding medical illness, psychiatric diagnosis, or recent (within 3 months) psychotropic substance history, recent (within 1 year) head trauma with loss of consciousness or functional sequelae, and confounding indwelling metal or conditions that would increase MRI risk. Additionally, all subjects were fluent in English. No significant past substance abuse/use disorder history (< 5-year lifetime total) was permitted, and urine toxicology screen was performed at the time of the first study visit.

As previously reported [14], genotyping was performed on Illumina genome-wide SNP chips (550 K–2.5 M SNPs). After performing genotype quality control procedures [49], phasing and imputation were performed using SHAPEIT and Impute2, from which SNPs rs710968, rs146777179, and rs6460071 genotypes were determined for each individual. Additionally, each participant was genotyped with the TaqMan 5′-exonuclease assay for *LIMK1* SNP rs710968, which showed 100% concordance with imputed genotypes for this SNP. PHASE software was used to determine 3-SNP haplotype groups. Individuals who were homozygous for all three major alleles (*GGC* 84.3%) were contrasted against individuals carrying a minor allele for any of these three SNPs. Predicted *LIMK1* expression levels were computed using previously reported methods [50]. After LD pruning, we used the score function of plink (version 1.9, https://www.cog-genomics.org/plink2) to weight each LD-independent SNP by the beta value from the *LIMK1* brain cortex cis-eQTL analysis from GTEx and create a transcription-based polygenic score for each individual. A *t*-test was then performed to determine whether the identified haplotype groups were associated with a difference in estimated *LIMK1* brain expression.

Structural MRIs were collected on a 1.5 Tesla GE scanner, using a T1-weighted SPGR sequence (*TR* = 24 ms, *TE* = 5 ms, flip angle 45°, 0.9375 × 0.9375 × 1.5-mm sagittal acquisition). SPM12’s DARTEL tool was used to create a group-specific template, which was then affine transformed into MNI space. Jacobian modulation maps computed from the DARTEL deformation field were applied to gray matter maps to produce gray matter volume maps. SPM12 was used to perform ANCOVA analysis on the haplotype groups, controlling for age, sex, and total brain size. Results referring to small volume correction (SVC) were obtained by performing family-wise error correction within an a priori volume defined by the original VBM findings for full-deletion WS participants in the IPS region, thresholded at *p* = 0.001. All participants gave written informed consent as part of protocols approved by National Institutes of Health Institutional Review Boards.

### General population study: PNC cohort

This replication cohort consisted of 255 healthy volunteers from the publicly available Philadelphia Neurodevelopment Cohort (PNC) [51] obtained from dbgap (accession number phs000607; mean age 16.0 ± 3.2 [std. dev.]; 125 males, 130 females). Participants were included in this analysis if they had the following: (i) a high-quality structural scan without evidence of significant artifacts based on visual inspection; (ii) high-quality genetic data from an Illumina SNP chip that clustered with the CEU and TSI HapMap3 populations, based on a principal components analysis of all genetic samples; and (iii) no significant past medical or neurological history. MRIs were collected on a 3-Tesla Siemens scanner as described elsewhere [51]. Genotyping, image processing and analysis were as described above for the NIMH GP cohort structural images. Polygenic-based scores for predicted *LIMK1* expression were also computed as above for the NIMH GP cohort.

## Results

### Structural and functional anomalies in children with Williams syndrome

**Fig. 1:**
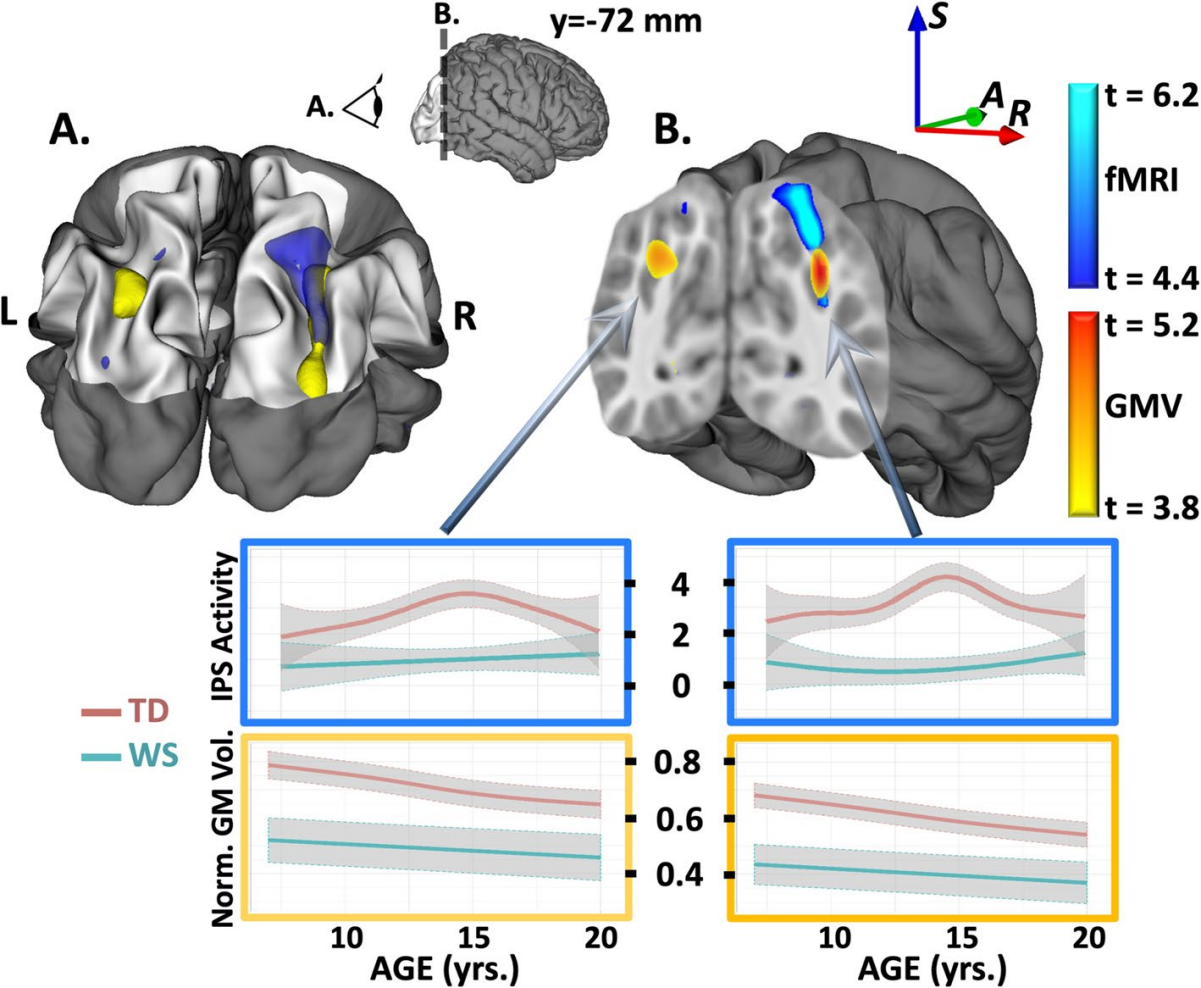
IPS findings in children with WS. Reduced gray matter volume (GMV in yellow/orange) and visuospatial-task-based BOLD activation (fMRI in blue) found in children with Williams syndrome (WS) relative to a group of typically developing (TD) children, both in initial cross-sectional analyses (GMV WS participants: *n* = 31, 21 F, age = 9.2 ± 3.2, TD participants: *n* = 64, 42 F, age = 9.3 ± 1.6; fMRI WS participants: *n* = 12, 11 F, age = 11.3 ± 2.9, TD participants *n* = 22, 9 F, age = 11.6 ± 2.5) and in the full longitudinal cohort (GMV WS participants: *n* = 33, 22 F, age = 12.0 ± 4.4, TD participants: *n* = 92, 56 F, age = 12.1 ± 3.1; fMRI WS participants: *n* = 15, 13 F, age = 12.6 ± 3.0, TD participants *n* = 34, 15 F, age = 13.3 ± 3.4). Initial cross-sectional findings for GMV and fMRI are shown here in 3D and coronal section, thresholded at *q* = 0.001 FDR and *q* = 0.01 FDR, respectively. Results for the full cohort (based on a linear mixed-effects approach) were similar. **A** Visualization of 3D extent of GMV and fMRI findings along the dorsal visual processing stream showing overlapping regions of structural and functional deficits in children with WS, particularly in the right hemisphere. **B** Visualization on the coronal plane (MNI *y* =  − 72 mm) showing spatial proximity of GMV/fMRI findings within/near IPS. Findings are consistent with previous findings in adults with WS [6]; spline-model developmental trajectories based on GMV and fMRI longitudinal cohorts — gray-shaded regions represent 95% confidence intervals

Our initial, cross-sectional study of children with WS (*N* = 31, ages 5–16 years) revealed structural and functional deficits in the IPS similar to those found in our previous adult studies, as shown in Fig. 1. Reductions in gray matter volume (MNI coordinates 31, − 72, 31, peak *t* = 5.1, *p* < 10^−4^ FDR corrected) overlapped with reductions in fMRI-based blood-oxygenation-level-dependent (BOLD) response (MNI coordinates 33, − 71, 29, peak *t* = 5.1, *p* < 0.05 FDR corrected) during performance of a custom-designed Tetris-like game that served as a child-friendly version of the shape-matching task used in our studies of adults with WS [6]. Reductions in gray matter volume and BOLD were bilateral within the IPS, though more pronounced in the right hemisphere.

### Longitudinal study of structural and functional anomalies in children with Williams syndrome

Our expanded, longitudinal study of children and young adults with WS (*N* = 33, 76 visits, ages 5–22 years) demonstrated that gray matter deficits within the IPS were essentially stable from the age of 5 years onward, results that are consistent with the presence of a genetically induced condition that remains stable throughout childhood, adolescence, and early adulthood. Linear mixed-effects analysis of structural MRI data from all longitudinal visits, based on a total of 1056 structural MRI scans (including typically developing controls), indicated gray matter deficits very similar to those found in our initial cross-sectional study, though more robust (*p* < 10^−6^ FDR corrected) given the additional statistical power. Longitudinal modeling of gray matter data across the brain, using a penalized splines approach [43], showed that, while gray matter within the IPS has a monotonically decreasing trajectory with age for both WS and typically developing groups, children with WS have a gray matter deficit that is relatively consistent over time. A similar longitudinal analysis of functional responses within the IPS to a visuospatial challenge (the Tetris-like fMRI task) showed consistently lower neural activation in children with WS relative to typically developing children, and also that children with WS lacked the increased response during the early teenage years that was demonstrated in typically developing children. The cross-sectional and longitudinal structural/functional findings and their developmental trajectories in children with WS are summarized in Fig. 1.

### Structural and functional anomalies in adults with WSCR short deletions

**Fig. 2:**
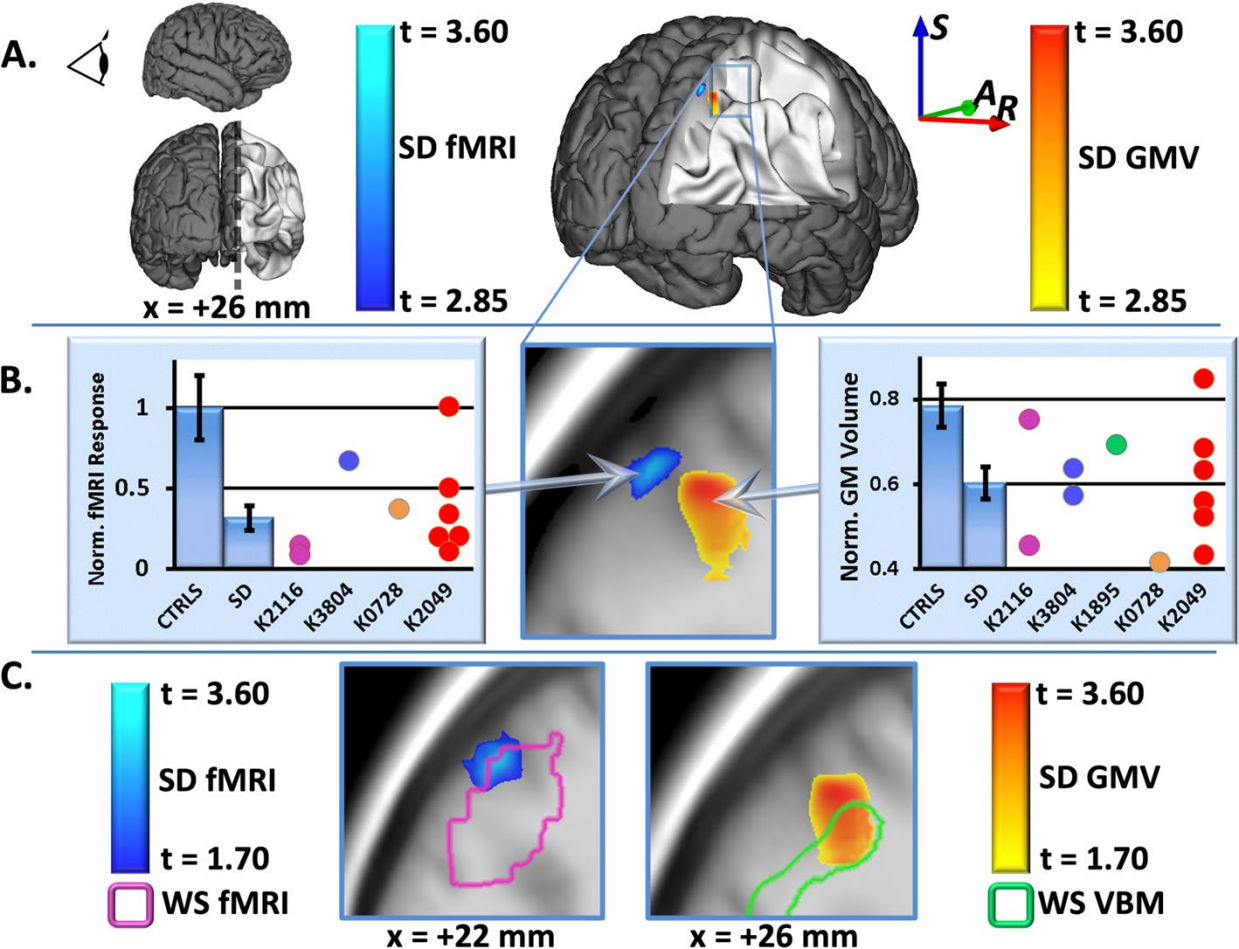
Structural and functional alterations in the short deletion (SD) group. **A** Visualization of the sagittal plane in the parietal cortex (dorsal visual processing stream) at *x* =  + 26 mm (MNI coordinate system — within the right IPS) showing results of gray matter analysis (yellow to red) demonstrating a local reduction in gray matter volume (GMV), accompanied by a nearby reduction in BOLD response observed during the square-completion fMRI task. fMRI results show a contrast between “square completion” and “matching” conditions, as described in the “Methods” section. Results are thresholded at *p* = 0.005. **B** Enlarged view of the sagittal plane shown in **A**, including plots of fMRI responses (left) and GMV (right) for the SD and control groups at the indicated locations. fMRI plot shows mean and SEM values for normalized beta coefficients found in the general linear model-based statistical analysis at the indicated location of maximum group difference (MNI coordinates 26, − 72, 51; *t* = 3.3; *p* = 0.002). Gray matter plot shows mean values and SEM for Jacobian-modulated gray matter volume at the point of maximum group difference (MNI coordinates 26, − 64, 47; *t* = 3.5; *p* = 0.001). **C** Illustration of degree of overlap between fMRI (left) and GMV (right) findings in the SD group and original corresponding fMRI and GMV findings [6] in full-deletion WS group. For these images, results are shown at a lower threshold of *p* = 0.05

Our study of adults with short deletions focused on a group of very rare individuals (*N* = 12, age = 35.4 ± 13.7 years) who have various short deletions that all include *LIMK1* (see Fig. S2 and additional clinical/genetic details previously reported [23]). We found structural and functional alterations within the right IPS (Fig. 2), with reduced gray matter volume (MNI coordinates 26, − 64, 47; peak *t* = 3.5; *p* = 0.001, uncorrected), as well as nearby reduction in BOLD response (MNI coordinates 26, − 72, 49; peak *t* = 3.3; *p* = 0.002, uncorrected) during a visuospatial square completion fMRI task (see “Methods”). These structural and functional alterations were observed in the context of normal task performance during fMRI (Table 4) but a reduction in this group’s visuospatial construction ability (*t* =  − 3.25, *p* = 0.006, uncorrected) as measured by formal, out-of-the-scanner neuropsychological examination of their performance (40.6 ± 2.75 [SEM]) on the block design portion (for which the mean standardized T-score for the general population is 50 ± 2.45 [SEM]) of the Wechsler Abbreviated Scale of Intelligence (WASI) [47].

### Haplotype-based structural variations in the general population

**Fig. 3:**
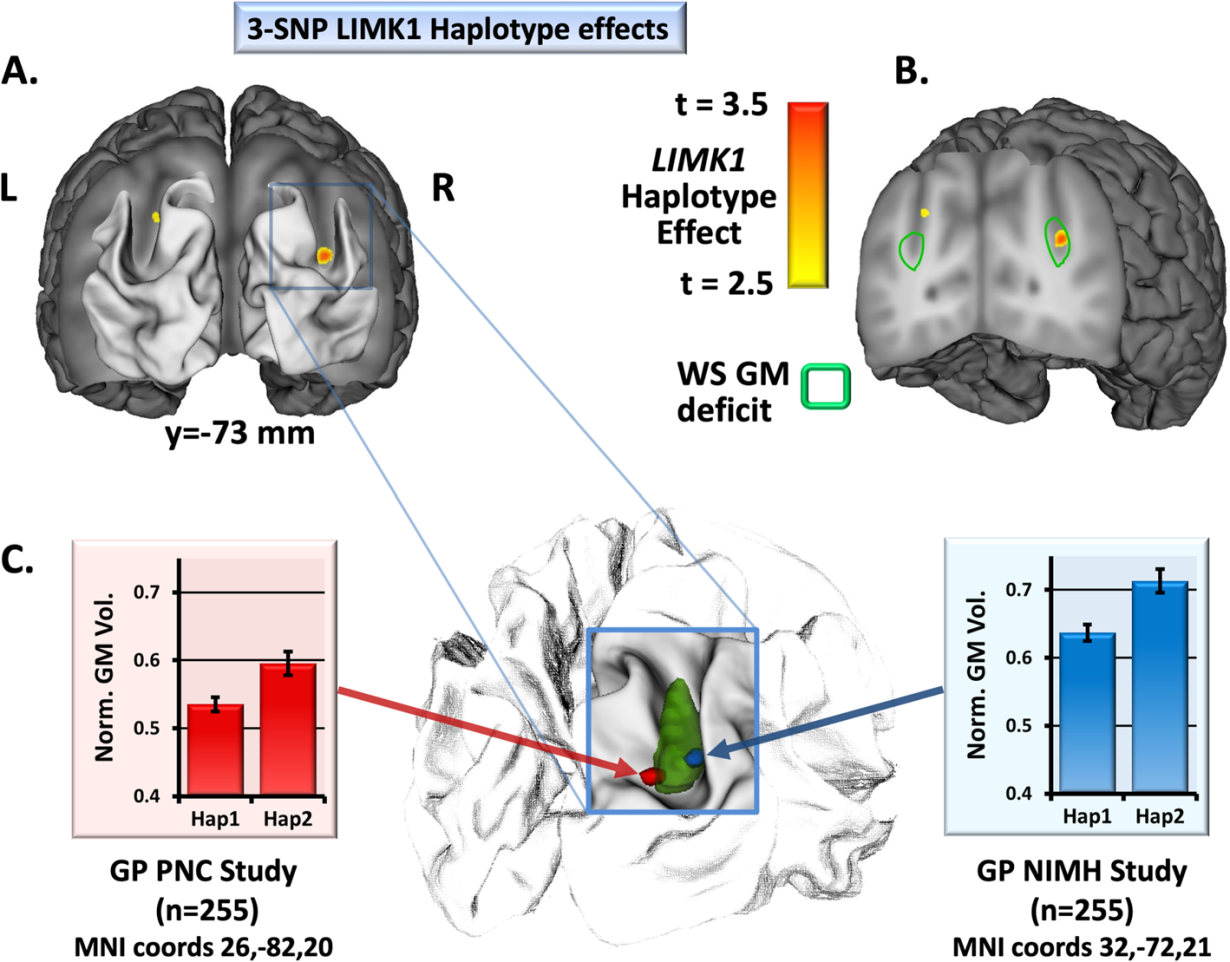
Reduced intraparietal sulcus (IPS) gray matter in the general population (GP) groups with *LIMK1* haplotype associated with reduced *LIMK1* expression. **A** Visualization of the coronal plane at *y* =  − 73 mm (MNI coordinate system — within the IPS) showing results of gray matter analysis demonstrating a local variation in gray matter volume (GMV) when comparing the lower-frequency haplotype group (Hap2) to the most common haplotype group (Hap1) in the NIMH GP study. Results are shown thresholded at *p* = 0.005. **B** Additional visualization of gray matter with haplotype on the coronal plane and congruent localization of GMV findings in the NIMH GP study relative to previous GMV findings (WS GMV deficit) in full-deletion WS [6]. Yellow-to-red coloring indicates the effect of variation in *LIMK1* haplotype in the NIMH GP study, while the green outline represents the extent of previous WS findings [6] at a threshold of *p* = 0.001 uncorrected. **C** Visualization of similar right IPS findings in two different GP cohorts. Blue and red regions show locations of haplotype variation within the right IPS in the NIMH and PNC GP cohorts, respectively, that are spatially coincident with the region of WS GMV deficit shown in green. Mean values and standard errors are shown for Jacobian-modulated GMV at the points of maximum group difference within the right IPS for the NIMH GP study (*t* = 3.73, *p* < 0.05 SVC) and the PNC GP study (*t* = 3.18, *p* = 0.0015, uncorrected)

For the general population studies of SNPs within the *LIMK1* gene, we studied structural MRI scans from two separate cohorts of healthy, right-handed Caucasian volunteers under 50 years of age. First, in 255 healthy, right-handed Caucasian volunteers (mean age = 32.6; 158 females) scanned at the NIMH, VBM analyses of gray matter distributions demonstrated that *LIMK1* haplotype variation, as described by Gregory et al. [14], predicted differences in IPS gray matter volume (MNI coordinates 33, − 73, 23, *t* = 3.73, *p* < 0.05 SVC, Fig. 3). The group of individuals with the most common haplotype (Hap1) had reduced gray matter volume within the IPS relative to the group with the less common haplotype (Hap2). Secondly, a confirmatory analysis of *LIMK1* haplotype effects was performed on a separate group of 255 healthy, right-handed Caucasian individuals (mean age = 16.0, 130 females) scanned as part of the PNC [51], and we found similar variations in gray matter volume (MNI coordinates 26, −82, 20, *t* = 3.18, *p* = 0.0015, uncorrected) within the right IPS in the same direction, as shown in Fig. 3C.

### Haplotype-based LIMK1 expression variations in the general population

**Fig. 4:**
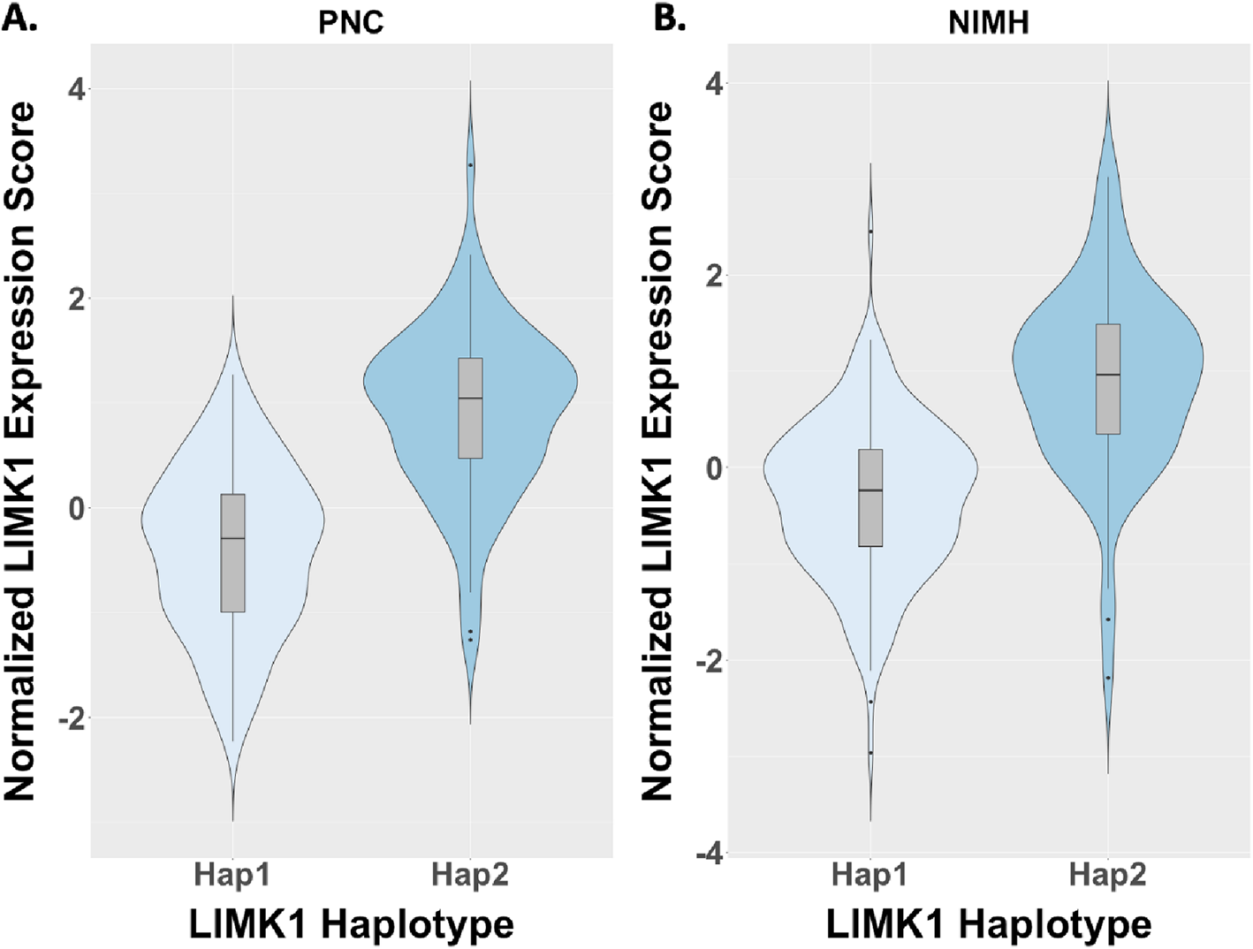
Variation in predicted LIMK1 expression with LIMK1 haplotype in the general population (GP) groups. Violin plots demonstrating that polygenic-based prediction of LIMK1 expression in the cortex, based on postmortem expression quantitative trait locus (eQTL) data obtained from the GTEx portal (https://gtexportal.org), was very strongly dependent on haplotype group in our two GP samples. Echoing the situation in the Williams syndrome hemideletion, the Hap1 group (with reduced IPS gray matter) had lower expression scores than the Hap2 group in both **A** the PNC sample (*N* = 255, *p* = 10^−23^) and **B** the NIMH sample (*N* = 255, *p* = 10^−15^)

To explore a potential *LIMK1*-based biological mechanism for the above findings, we took advantage of available gene-expression data to ask whether the two haplotype groups within the general population might have different levels of relevant *LIMK1* expression. Although we have previously shown that individual SNPs comprising the haplotype are associated with *LIMK1* expression in the postmortem brain [14], we sought to add confirmatory evidence to this finding using our living general population cohorts. Since gene expression cannot be readily measured in the brains of living individuals directly, we employed a method to estimate *LIMK1* brain expression for each individual [50], based on postmortem expression quantitative trait locus (eQTL) data for brain cortex obtained from the GTEx portal (https://gtexportal.org). We found that, in both general population cohorts, polygenic-based prediction of *LIMK1* expression in the cortex was very strongly dependent on haplotype group in a manner that echoed the situation in Williams syndrome itself [52]: the Hap1 group (with reduced IPS gray matter) had lower estimated expression scores (*p* = 10^−15^ for the NIMH group, *p* = 10^−23^ for the PNC group) than the Hap2 group (see Fig. 4). These results are in accordance with variations in LIMK1 expression based on SNPs that make up the haplotype in postmortem GTEx samples of cortex, as depicted in Gregory et al. (Supplementary Figure S6) [14].

## Discussion

Our results in children with Williams syndrome demonstrate that structural and functional IPS anomalies are present in early childhood, and the longitudinal data demonstrate that these are stable across development into early adulthood, consistent with an enduring genetic mechanism. The results of both the short deletion and the two general population studies support the hypothesis that *LIMK1* plays an important role in the structural and functional development of the IPS dorsal stream region — specifically that these observations may be the downstream neurostructural and neurofunctional effects of differences (induced by either allelic variation as in the general population or haploinsufficiency as is the case in Williams syndrome and the participants with short deletions) in the function of LIMK1 protein. These differences may have influenced cellular migration (via modulation of actin) in a particular manner and/or during a particular early developmental time window when the IPS is vulnerable.

In the case of the short deletion study, we showed structural and functional alterations within the IPS, in combination with a neuropsychologically measured deficit in visuospatial construction, consistent with the hypothesis that, despite the heterogeneity of the various kindreds’ hemideletions, their shared *LIMK1* haploinsufficiency was sufficient to affect not only this cognitive ability but also neuroanatomical and neurostructural neurodevelopment processes within the IPS. These results also imply that deletions of telomerically located genes within the WS 7q11.23 locus, including the general transcription factor genes *GTF2I* and *GTF2IRD1*, are not required to produce the structural, functional, or behavioral deficits described here (since these genes were intact for all participants with short deletions), which is consistent with recent mouse studies of these two genes [53].

Importantly, our findings do not exclude the likely possibility of interactions between alterations in *LIMK1* and *GTF2I* (involved in multiple relevant gene networks [54] and especially implicated in myelination processes) [21] or other genes, such as *CLIP2* (which codes for a microtubule binding protein), which could affect the magnitude of these brain findings and the visuospatial construction deficit. Indeed, the effect sizes (values of Cohen’s d) [55] for the participants with short deletions (for IPS gray matter volume 1.18, BOLD signal change during square completion 1.28, and block design performance 0.99), while large, were smaller than previously reported [6] for participants with WS who have typical full 7q11.23 hemideletions (2.67, 1.64, and 1.50, respectively). Moreover, there is a strong biological basis for a hypothetical interaction between *LIMK1* and *CLIP2* based on their roles in cytoskeleton dynamics [31, 56]. Other WSCR genes may play interactive roles as well [54]. Recent work by Tebbenkamp et al. [19] provides evidence that *DNAJC30* contributes to Williams syndrome pathogenesis via a mitochondrial-based mechanism, but also implicates *LIMK1* as a potential contributor, since *DNAJC30* and *LIMK1* are identified as part of a shared functional cluster of genes, based on co-expression and protein-to-protein interaction networks.

Our results in two separate cohorts in the general population studies demonstrate that haplotype variations in *LIMK1* have an impact on brain structure that is reminiscent, in both form and location, of the structural differences found in Williams syndrome and are consistent with previous findings of *LIMK1* haplotype-based variations in functional connectivity within the same IPS region [14]. It is important to note the genetic context of the general population study results — i.e., no relevant genes are deleted, and the *LIMK1* variations we have investigated are common among healthy individuals who do not have Williams syndrome. Nevertheless, the combination of results with respect to brain structure and expression levels implies that haplotype-mediated expression of *LIMK1* can have early developmental impacts that specifically affect gray matter volume within the IPS.

Although we observed differences in gray matter volume, it is possible that the integrity of white matter structure plays a primary role during critical development periods, since Van Essen’s tension-based theory [30] would imply that early development of tension-mediated cortical folding patterns could be directly affected by reductions in the structural integrity of white matter fibers. It is worth noting that, while mouse models have proven to be valuable in the study of Williams syndrome [57], the genetic mechanism hypothesized here might not be observable in the case of the lissencephalic mouse brain, thus limiting the utility of mouse models and advocating for human and nonhuman primate studies of this particular genotype–phenotype link.

## Conclusions

Taken together, these results provide evidence that variation in *LIMK1* plays an important role in both the WS phenotype and typical development of the dorsal stream region. The hypothesis-driven studies presented here provide evidence that *LIMK1* variations produce concomitant variations in observable brain phenotypes, presumably as a result of molecular processes affecting neuronal migration, which affect structure and function of the dorsal visual processing stream and have relevance for the hallmark cognitive deficit in Williams syndrome. More generally, these data provide a striking example of the mechanisms by which genetic variation, acting by means of molecular effects on a neural intermediary, can influence human cognition and, in some cases, lead to neurocognitive disorders.

### Supplementary Information

**Additional file 1: Figure S1.** “Spaghetti plots” illustrating timelines of participant visits for longitudinal studies of children with Williams syndrome and typically developing participants. **Figure S2.** Schematic diagram depicting locations of Williams syndrome critical region hemizygous deletions in short deletion kindreds described in this study.

## Data Availability

NIMH patient data are not publicly available due to institutional (IRB) restrictions. NIMH general population imaging data are in the process of being publicly shared as permitted via IRB approval and participant consents. PNC imaging and genetic info are available via dbGaP (study accession ID phs000607.v1.p1).

## Abbreviations

AFNI: Analysis of Functional Neuroimages (software)
FWHM: Full-width half maximum
GP: General population
IPS: Intraparietal sulcus
NIMH: National Institute of Mental Health
PNC: Philadelphia Neurodevelopmental Cohort
SD: Short deletion
SNP: Single-nucleotide polymorphism
SPM: Statistical parametric mapping (software)
SVC: (Family-wise) small volume correction
TD: Typically developing
WS: Williams syndrome

## References

[1] Bayes M, Magano LF, Rivera N, Flores R, Perez Jurado LA (2003). Mutational mechanisms of Williams-Beuren syndrome deletions. Am J Hum Genet.

[2] Mervis CB, Robinson BF, Bertrand J, Morris CA, Klein-Tasman BP, Armstrong SC (2000). The Williams syndrome cognitive profile. Brain Cogn.

[3] Frangiskakis JM, Ewart AK, Morris CA, Mervis CB, Bertrand J, Robinson BF (1996). LIM-kinase1 hemizygosity implicated in impaired visuospatial constructive cognition. Cell.

[4] Mervis CB, John AE (2010). Cognitive and behavioral characteristics of children with Williams syndrome: implications for intervention approaches. Am J Med Genet C Semin Med Genet.

[5] Ungerleider LG, Mishkin M. Two cortical visual systems. In: Ingle DJ, DJG, Mansfield RJW, editors. Cambridge: The MIT Press; 1982.

[6] Meyer-Lindenberg A, Kohn P, Mervis CB, Kippenhan JS, Olsen RK, Morris CA (2004). Neural basis of genetically determined visuospatial construction deficit in Williams syndrome. Neuron.

[7] Kippenhan JS, Olsen RK, Mervis CB, Morris CA, Kohn P, Meyer-Lindenberg A (2005). Genetic contributions to human gyrification: sulcal morphometry in Williams syndrome. J Neurosci.

[8] Van Essen DC, Dierker D, Snyder AZ, Raichle ME, Reiss AL, Korenberg J (2006). Symmetry of cortical folding abnormalities in Williams syndrome revealed by surface-based analyses. J Neurosci.

[9] Fahim C, Yoon U, Nashaat NH, Khalil AK, El-Belbesy M, Mancini-Marie A (2012). Williams syndrome: a relationship between genetics, brain morphology and behaviour. J Intellect Disabil Res.

[10] Boddaert N, Mochel F, Meresse I, Seidenwurm D, Cachia A, Brunelle F (2006). Parieto-occipital grey matter abnormalities in children with Williams syndrome. Neuroimage.

[11] Meyer-Lindenberg A, Mervis CB, Berman KF (2006). Neural mechanisms in Williams syndrome: a unique window to genetic influences on cognition and behaviour. Nat Rev Neurosci.

[12] Gagliardi C, Arrigoni F, Nordio A, De Luca A, Peruzzo D, Decio A (2018). A different brain: anomalies of functional and structural connections in Williams syndrome. Front Neurol.

[13] Vega JN, Hohman TJ, Pryweller JR, Dykens EM, Thornton-Wells TA (2015). Resting-state functional connectivity in individuals with Down syndrome and Williams syndrome compared with typically developing controls. Brain Connect.

[14] Gregory MD, Mervis CB, Elliott ML, Kippenhan JS, Nash T, Czarapata JB (2019). Williams syndrome hemideletion and LIMK1 variation both affect dorsal stream functional connectivity. Brain.

[15] Tranel D, Vianna E, Manzel K, Damasio H, Grabowski T (2009). Neuroanatomical correlates of the Benton Facial Recognition Test and Judgment of Line Orientation Test. J Clin Exp Neuropsychol.

[16] Faillenot I, Decety J, Jeannerod M (1999). Human brain activity related to the perception of spatial features of objects. Neuroimage.

[17] Kitada R, Kito T, Saito DN, Kochiyama T, Matsumura M, Sadato N (2006). Multisensory activation of the intraparietal area when classifying grating orientation: a functional magnetic resonance imaging study. J Neurosci.

[18] Kozel BA, Barak B, Kim CA, Mervis CB, Osborne LR, Porter M (2021). Williams syndrome. Nat Rev Dis Primers.

[19] Tebbenkamp ATN, Varela L, Choi J, Paredes MI, Giani AM, Song JE (2018). The 7q11.23 protein DNAJC30 interacts with ATP synthase and links mitochondria to brain development. Cell.

[20] Chailangkarn T, Trujillo CA, Freitas BC, Hrvoj-Mihic B, Herai RH, Yu DX (2016). A human neurodevelopmental model for Williams syndrome. Nature.

[21] Barak B, Zhang Z, Liu Y, Nir A, Trangle SS, Ennis M (2019). Neuronal deletion of Gtf2i, associated with Williams syndrome, causes behavioral and myelin alterations rescuable by a remyelinating drug. Nat Neurosci.

[22] Chailangkarn T, Noree C, Muotri AR (2018). The contribution of GTF2I haploinsufficiency to Williams syndrome. Mol Cell Probes.

[23] Morris CA, Mervis CB, Hobart HH, Gregg RG, Bertrand J, Ensing GJ (2003). GTF2I hemizygosity implicated in mental retardation in Williams syndrome: genotype-phenotype analysis of five families with deletions in the Williams syndrome region. Am J Med Genet A.

[24] Gao MC, Bellugi U, Dai L, Mills DL, Sobel EM, Lange K (2010). Intelligence in Williams syndrome is related to STX1A, which encodes a component of the presynaptic SNARE complex. PLoS One.

[25] Proschel C, Blouin MJ, Gutowski NJ, Ludwig R, Noble M (1995). Limk1 is predominantly expressed in neural tissues and phosphorylates serine, threonine and tyrosine residues in vitro. Oncogene.

[26] Ghosh M, Song X, Mouneimne G, Sidani M, Lawrence DS, Condeelis JS (2004). Cofilin promotes actin polymerization and defines the direction of cell motility. Science.

[27] Dong Q, Ji YS, Cai C, Chen ZY (2012). LIM kinase 1 (LIMK1) interacts with tropomyosin-related kinase B (TrkB) and mediates brain-derived neurotrophic factor (BDNF)-induced axonal elongation. J Biol Chem.

[28] Hu K, Ji L, Applegate KT, Danuser G, Waterman-Storer CM (2007). Differential transmission of actin motion within focal adhesions. Science.

[29] Rakic P (1988). Specification of cerebral cortical areas. Science.

[30] Van Essen DC (1997). A tension-based theory of morphogenesis and compact wiring in the central nervous system. Nature.

[31] Hoogenraad CC, Akhmanova A, Galjart N, De Zeeuw CI (2004). LIMK1 and CLIP-115: linking cytoskeletal defects to Williams syndrome. BioEssays.

[32] Meng Y, Zhang Y, Tregoubov V, Janus C, Cruz L, Jackson M (2002). Abnormal spine morphology and enhanced LTP in LIMK-1 knockout mice. Neuron.

[33] Todorovski Z, Asrar S, Liu J, Saw NM, Joshi K, Cortez MA (2015). LIMK1 regulates long-term memory and synaptic plasticity via the transcriptional factor CREB. Mol Cell Biol.

[34] Akagawa H, Tajima A, Sakamoto Y, Krischek B, Yoneyama T, Kasuya H (2006). A haplotype spanning two genes, ELN and LIMK1, decreases their transcripts and confers susceptibility to intracranial aneurysms. Hum Mol Genet.

[35] Gray V, Karmiloff-Smith A, Funnell E, Tassabehji M (2006). In-depth analysis of spatial cognition in Williams syndrome: a critical assessment of the role of the LIMK1 gene. Neuropsychologia.

[36] Hamer D (2002). Genetics. Rethinking behavior genetics. Science.

[37] Sled JG, Zijdenbos AP, Evans AC (1998). A nonparametric method for automatic correction of intensity nonuniformity in MRI data. IEEE Trans Med Imaging.

[38] Cox RW (1996). AFNI: software for analysis and visualization of functional magnetic resonance neuroimages. Comput Biomed Res.

[39] Ashburner J, Ridgway GR (2012). Symmetric diffeomorphic modeling of longitudinal structural MRI. Front Neurosci.

[40] Whitfield-Gabrieli S, Nieto-Castanon A (2012). Conn: a functional connectivity toolbox for correlated and anticorrelated brain networks. Brain Connect.

[41] Avants B, Khan A, McCluskey L, Elman L, Grossman M (2009). Longitudinal cortical atrophy in amyotrophic lateral sclerosis with frontotemporal dementia. Arch Neurol.

[42] Wood SN (2017). Generalized additive models: an introduction with R.

[43] Chen G, Nash TA, Cole KM, Kohn PD, Wei SM, Gregory MD (2021). Beyond linearity in neuroimaging: capturing nonlinear relationships with application to longitudinal studies. Neuroimage.

[44] Chen G, Saad ZS, Britton JC, Pine DS, Cox RW (2013). Linear mixed-effects modeling approach to FMRI group analysis. Neuroimage.

[45] Pani AM, Hobart HH, Morris CA, Mervis CB, Bray-Ward P, Kimberley KW (2010). Genome rearrangements detected by SNP microarrays in individuals with intellectual disability referred with possible Williams syndrome. PLoS One.

[46] Ashburner J (2007). A fast diffeomorphic image registration algorithm. Neuroimage.

[47] Wechsler D (1999). Wechsler abbreviated scale of intelligence.

[48] Missar CD, Gold JM, Goldberg TE (1994). WAIS-R short forms in chronic schizophrenia. Schizophr Res.

[49] Anderson CA, Pettersson FH, Clarke GM, Cardon LR, Morris AP, Zondervan KT (2010). Data quality control in genetic case-control association studies. Nat Protoc.

[50] Gamazon ER, Wheeler HE, Shah KP, Mozaffari SV, Aquino-Michaels K, Carroll RJ (2015). A gene-based association method for mapping traits using reference transcriptome data. Nat Genet.

[51] Satterthwaite TD, Elliott MA, Ruparel K, Loughead J, Prabhakaran K, Calkins ME (2014). Neuroimaging of the Philadelphia neurodevelopmental cohort. Neuroimage.

[52] Kimura R, Swarup V, Tomiwa K, Gandal MJ, Parikshak NN, Funabiki Y (2019). Integrative network analysis reveals biological pathways associated with Williams syndrome. J Child Psychol Psychiatry.

[53] Kopp N, McCullough K, Maloney SE, Dougherty JD (2019). Gtf2i and Gtf2ird1 mutation do not account for the full phenotypic effect of the Williams syndrome critical region in mouse models. Hum Mol Genet.

[54] Chen L, Wang W, Cai W, Song W, Qian W, Lin GN (2021). Spatiotemporal 7q11.23 protein network analysis implicates the role of DNA repair pathway during human brain development. Sci Rep.

[55] Cohen J (1992). A power primer. Psychol Bull.

[56] Hoogenraad CC, Akhmanova A (2010). Dendritic spine plasticity: new regulatory roles of dynamic microtubules. Neuroscientist.

[57] Osborne LR (2010). Animal models of Williams syndrome. Am J Med Genet C Semin Med Genet.

