# Supplementary material for: Dorsal visual stream and LIMK1: hemideletion, haplotype, and enduring effects in children with Williams syndrome
Source: J Neurodev Disord. 2023 Aug 26;15:29. doi: 10.1186/s11689-023-09493-x (PMC10464045; doi:10.1186/s11689-023-09493-x)
Supplement: Supplementary file 1 — Additional file 1: Figure S1. “Spaghetti plots” illustrating timelines of participant visits for longitudinal studies of children with Williams syndrome and typically developing participants. Figure S2. Schematic diagram depicting locations of Williams syndrome critical region hemizygous deletions in short deletion kindreds described in this study. [file 11689_2023_9493_MOESM1_ESM.docx]

**Supplementary Materials:**

Figures S1-S2


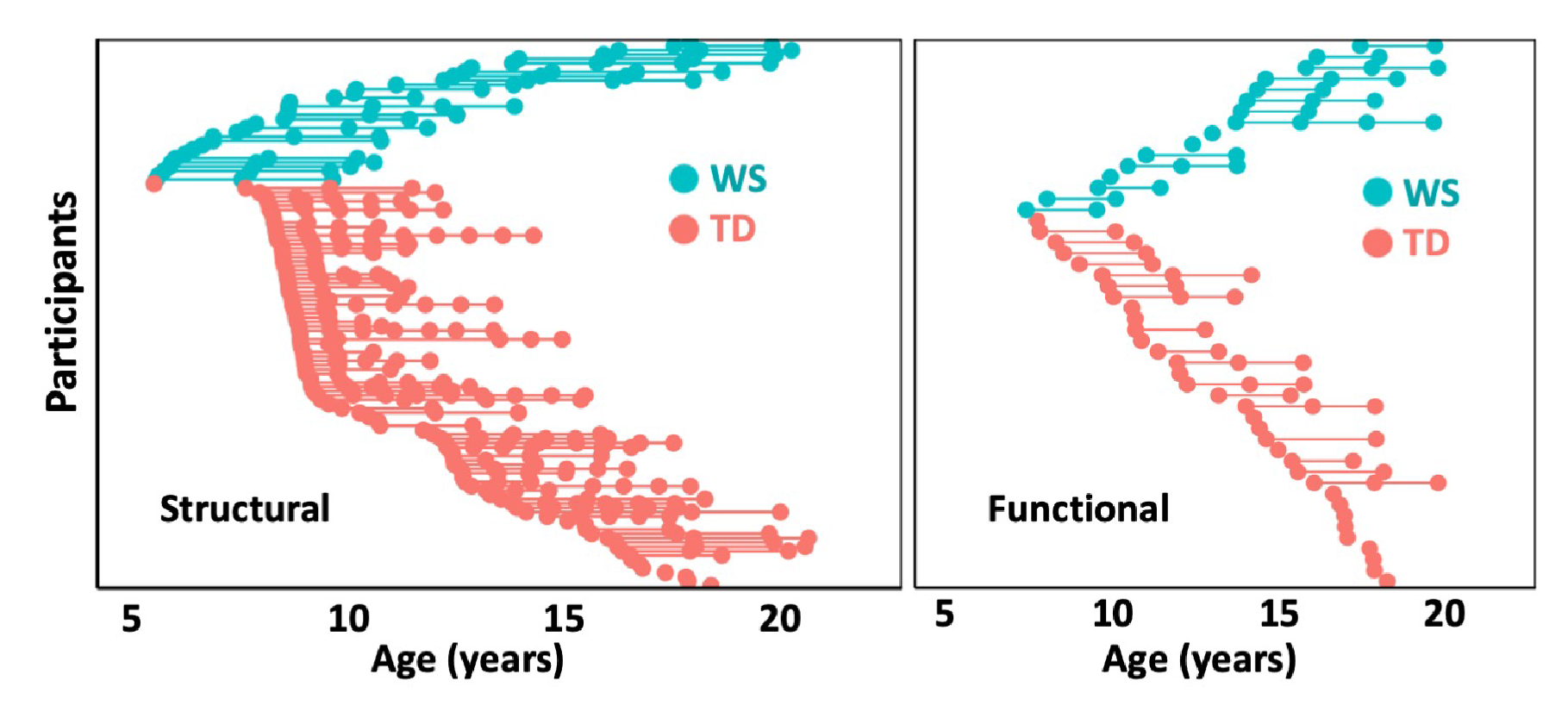


**Figure S1**. “Spaghetti plots” illustrating timelines of participant visits for longitudinal structural (left) and functional (right) studies of children with Williams syndrome (teal) and typically developing participants (red). Each horizontal line represents a participant, each circle represents a visit, and connected circles represent visits for the same participant.


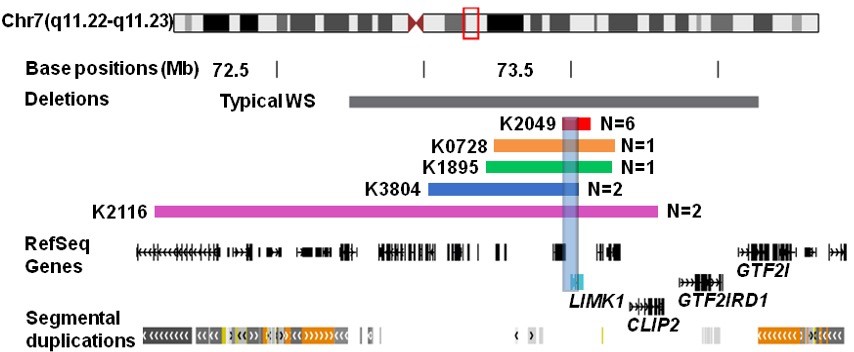


**Figure S2.** Schematic diagram depicting locations of Williams syndrome critical region hemizygous deletions in SD kindreds described in this study on the hg19 human genome assembly. Red box on ideogram shows region of detail. Grey bar shows the typical WS deletion caused by non-allelic homologous recombination between flanking segmental duplications. Colored horizontal bars denote locations of atypical deletions in study kindreds, with numbers indicating participants per kindred. Transparent blue vertical bar shows the shared region deleted in all study participants, which includes only *ELN* and *LIMK1*. Refseq genes^1, 2^ with hypothesized specific contributions to WS neural phenotypes are labeled, and others are collapsed into a single track. *LIMK1* is depicted in aqua. Figure adapted from the UCSC genome browser.^3^

1. Dalgleish R, Flicek P, Cunningham F, Astashyn A, Tully RE, Proctor G, et al. Locus Reference Genomic sequences: an improved basis for describing human DNA variants. Genome Med. 2010;2(4):24.

2. Pruitt KD, Tatusova T, Maglott DR. NCBI reference sequences (RefSeq): a curated non-redundant sequence database of genomes, transcripts and proteins. Nucleic Acids Res. 2007;35(Database issue):D61-5.

3. Kent WJ, Sugnet CW, Furey TS, Roskin KM, Pringle TH, Zahler AM, et al. The human genome browser at UCSC. Genome Res. 2002;12(6):996-1006.
